# microRNA-378a-5p iS a novel positive regulator of melanoma progression

**DOI:** 10.1038/s41389-020-0203-6

**Published:** 2020-02-14

**Authors:** Maria Grazia Tupone, Simona D’Aguanno, Marta Di Martile, Elisabetta Valentini, Marianna Desideri, Daniela Trisciuoglio, Sara Donzelli, Andrea Sacconi, Simonetta Buglioni, Cristiana Ercolani, Alessio Biagioni, Gabriella Fibbi, Luigi Fattore, Rita Mancini, Gennaro Ciliberto, Giovanni Blandino, Donatella Del Bufalo

**Affiliations:** 1 Preclinical Models and New Therapeutic Agents Unit, IRCCS Regina Elena National Cancer Institute, Rome, Italy; 2 Institute of Molecular Biology and Pathology, National Research Council, Rome, Italy; 3 Oncogenomics and Epigenetics Unit, IRCCS Regina Elena National Cancer Institute, Rome, Italy; 4 Clinical Trial Center, Biostatistics and Bioinformatics Unit, IRCCS Regina Elena National Cancer Institute, Rome, Italy; 5 Pathology Unit, IRCCS Regina Elena National Cancer Institute, Rome, Italy; 6 Department of Experimental and Clinical Biomedical Sciences “Mario Serio”, University of Florence, Florence, Italy; 7 Department of Melanoma, Oncologic Immunotherapy and Innovative Therapies, Istituto Nazionale Tumori IRCCS, “Fondazione G. Pascale”, Naples, Italy; 8 Department of Molecular and Clinical Medicine, Risk Management Q&A, Sant’Andrea Hospital, “Sapienza” University, Rome, Italy; 9 Scientific Direction, IRCCS Regina Elena National Cancer Institute, Rome, Italy; 10 Present Address: Department of Life, Health and Environmental Sciences, University of L’Aquila, L’Aquila, Italy

**Keywords:** Cell biology, Skin cancer

## Abstract

Evaluating the expression levels of miR-378a-5p both in a large melanoma patient cohort from The Cancer Genome Atlas database and in melanoma patients from our Institute, we found that miR-378a-5p is upregulated in metastatic melanoma specimens. miR-378a-5p expression was also increased in melanoma cells resistant to target therapy, and decreased in response to drug treatment. We also demonstrated that overexpression of miR-378a-5p enhances in vitro cell invasion and migration, and facilitates the ability of melanoma cells to form de novo vasculogenic structures. While performing downstream targeting studies, we confirmed the ability of miR-378a-5p to modulate the expression of known target genes, such as SUFU, FUS-1, and KLF9. Luciferase-3′UTR experiments also identified STAMBP and HOXD10 as new miR-378a-5p target genes. MMP2 and uPAR, two HOXD10 target genes, were positively regulated by miR-378a-5p. Genetic and pharmacologic approaches inhibiting uPAR expression and activity evidenced that the in vitro tumor-promoting functions of miR-378a-5p, were in part mediated by uPAR. Of note miR-378a-5p was also able to increase VEGF, as well as in vitro and in vivo angiogenesis. Finally, genetic and pharmacologic modulation of Bcl-2 evidenced Bcl-2 ability to regulate miR-378a-5p expression. In conclusion, to the best of our knowledge, this is the first study demonstrating that miR-378a-5p acts as an oncogenic microRNA in melanoma.

## Introduction

Understanding the molecular mechanisms behind melanoma is crucial to identify key regulators of its progression. Increasing evidence identified dysregulation of microRNAs (miRNA) expression in melanoma tissues and in peripheral blood of melanoma patients^1^. Several miRNA have been identified as the main players in melanoma dissemination and response to therapy, acting either as tumor suppressors or oncogenes^1^. In this context, we previously demonstrated that miR-211 plays a role in Bcl-2-induced melanoma promoting functions^2^, miR-579-3p controls melanoma progression and resistance to target therapy^3^, and miR-25 is involved in the regulation of PTEN expression by MEK inhibition^4^.

The current study aimed at investigating the biological and functional role of miR-378a-5p in melanoma. Together with miR-378a-3p, miR-378a-5p is one of the two mature strands of miR-378a, previously known as miR-378^5^. While miR-378a-3p has been reported to positively regulate epithelial mesenchymal transition and metastasis of melanoma^6^, and edited miR-378a-3p, but not its wild-type form, has been found to inhibit melanoma metastatization^7^, to the best of our knowledge no data have been published so far on the relevance of miR-378a-5p in melanoma progression. miR-378a-5p plays a regulatory role in drugs/toxins and oxidative metabolism, angiogenesis, cardiovascular system and muscle biology^5^. In the last two decades, the involvement of miR-378a-5p in tumor angiogenesis and progression has also been revealed. miR-378a-5p has been found downregulated in gastric^8^, oral^9^, and colon carcinoma^10^, and upregulated in renal carcinoma^11^ and in acute myeloid leukemia^12^. Moreover, miR-378a-5p enhances cell survival and promotes glioblastoma and non-small cell lung cancer growth and angiogenesis^13,14^, perturbs mitotic fidelity and correlates with breast cancer progression^15^ and choriocarcinoma differentiation^16^, positively affects tumor formation by delaying oncogene-induced senescence^17^, promotes invasion and ovarian estradiol production^18^ and induces mesenchymal stem cell vascularization and survival^19^. The role of miR-378a-5p as tumor suppressor gene in the carcinogenesis of colorectal and renal carcinoma has been also evidenced^20,21^. Studies in serum or plasma of cancer patients indicate miR-378a-5p as a potential biomarker^11,12^.

## Results

### miR-378a-5p expression correlates with melanoma progression, drug resistance and response to therapy

**Fig. 1:**
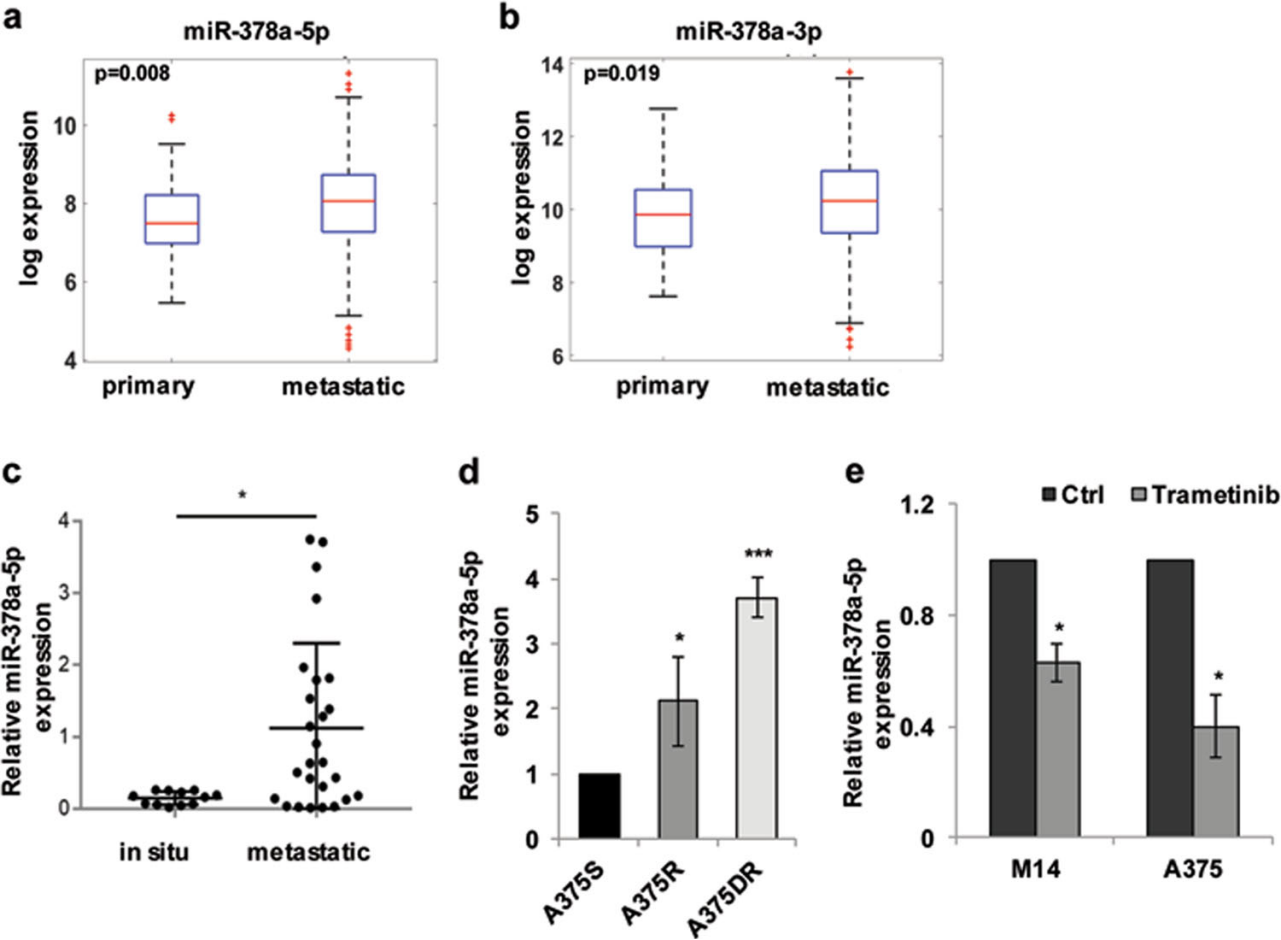
miR-378a-5p expression correlates with melanoma progression and drug resistance. **a**, **b** Expression levels of **a** miR-378a-5p and **b** miR-378a-3p in a melanoma patient cohort, including 351 metastatic and 96 primary melanoma specimens, from The Cancer Genome Atlas database (TCGA) (http://cancergenome.nih.gov/). **c** qRT-PCR analysis of miR-378a-5p expression in 27 cases of metastatic melanoma specimens and in 13 specimens from in situ melanoma lesions collected at Regina Elena National Cancer Institute/San Gallicano Dermatologic Institute (Istituti Fisioteparici Ospitalieri-IFO) *p *<* *0.05*. **a**–**c** Statistical analysis was performed applying Mann-Whitney test. **d** Expression levels of miR-378a-5p in A375 cells sensitive (A375S), resistant to Vemurafenib (A375R) or to Dabrafenib/Trametinib (A375DR). The results are reported as ratio of resistant cells relative to sensitive ones ± SEM. **e** Expression levels of miR-378a-5p in M14 and A375 cells untreated (Ctrl) or treated with Trametinib (0.001 μM, 72 h). The results are reported as ratio of treated cells relative to control ones ± SEM. **d**, **e** Statistical analysis was performed applying *t*-test, *p < 0.05; ***p < 0.001.

We firstly evaluated the expression levels of miR-378a-5p and miR-378a-3p in a large melanoma patient cohort from The Cancer Genome Atlas (TCGA) database. As shown in Fig. 1a, b, both miRNAs were significantly upregulated in metastatic melanoma when compared to primary melanoma. Motivated by the lack of any data on the role of miR-378a-5p in melanoma pathobiology, we focused our attention on the biological functions of this miRNA in human melanoma. As reported in Fig. 1c, the expression of miR-378a-5p was significantly higher in melanoma specimens from our Institute compared to melanoma in situ. We also found an increased miR-378a-5p expression in the A375 melanoma cells resistant to Vemurafenib (BRAF inhibitor) or to Dabrafenib/Trametinib (BRAF/MEK inhibitors)^22^ compared to sensitive ones (Fig. 1d). Interestingly, while exposure to Dabrafenib did not affect miR-378a-5p expression (data not shown), the treatment with Trametinib significantly reduced its expression in both M14 and A375 cells (Fig. 1e).

Next, we evaluated the effect of miR-378a-5p on the expression of some target genes (Supplementary Table 1) including putative, such as STAMBP and SP1, or validated, such as FUS-1/TUSC2, SUFU and KLF9^14,19,23^. To this purpose we modulated miR-378a-5p expression transfecting melanoma cells with hsa-miR-378a-5p miRNA mimic or antisense sequence against miR-378 or the relative scrambled controls. Upregulation of miR-378a-5p in M14 melanoma cells reduced both mRNA (Supplementary Fig. 1a) and protein (Supplementary Fig. 1b) levels of STAMBP, SP1, KLF9, FUS-1, and SUFU when compared with control transfected cells, while miR-378a-5p inhibition led to an opposite effect, upregulating the expression of target genes (Supplementary Fig. 1c). As reported in Supplementary Fig. 1d, luciferase assay confirmed the binding of miR-378a-5p to the 3′UTR region of STAMBP mRNA.

### miR-378a-5p promotes in vitro tumor-progression-associated properties and the expression of vascular endothelial growth factor (VEGF) and urokinase-type plasminogen receptor (uPAR)

We next assessed the functional relevance of miR-378a-5p on in vitro cell proliferation, migration, invasion, and clonogenic ability. Contrary to the results demonstrating miR-378a-5p ability to affect proliferation of several tumor histotypes^23,24^, we did not observe any effect of miR-378a-5p either on proliferation or clonogenic ability of M14 melanoma cells (Supplementary Fig. 2).

**Fig. 2:**
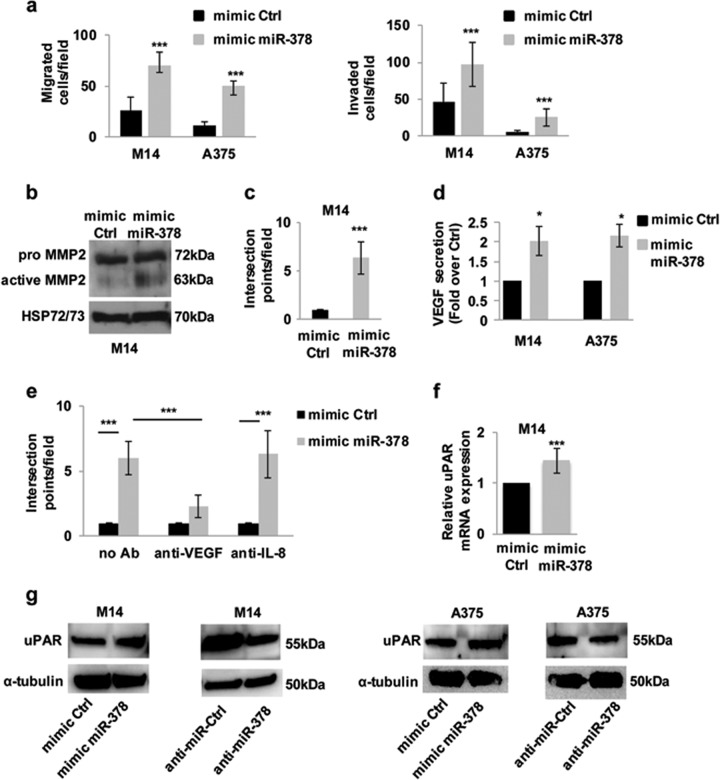
miR-378a-5p (here abbreviated to miR-378) promotes in vitro tumor-progression-associated properties and induces uPAR expression in melanoma cells. **a** In vitro cell migration and invasion of M14 and A375 melanoma cells transiently transfected with mimic miRNA scramble control (mimic Ctrl) or mimic miR-378. Values are expressed as number of migrated/invaded cells ± standard deviation. **b** Western blot analysis of MMP2 protein expression in M14 cells transiently transfected with mimic Ctrl or mimic miR-378. **c** Quantification of capillary-like structures formation in M14 cells transiently transfected with mimic Ctrl or mimic miR-378. Values are expressed as number of intersection points/field ± standard deviation. **d** VEGF secretion by M14 and A375 cells transfected with mimic Ctrl or mimic miR-378. Results are reported as fold over control. **e** Quantification of capillary-like structures formation in M14 cells transiently transfected with mimic Ctrl or mimic miR-378 incubated in the absence (no Ab) or presence of specific human VEGF- (anti-VEGF) or IL-8- (anti-IL-8) -neutralizing antibodies (0.2 ug/mL). Results are reported as number of intersection points/field (average ± SD). **f** qRT-PCR analysis of uPAR mRNA expression in M14 cells transfected with mimic Ctrl or mimic miR-378. The results are reported as fold induction ± SEM in cells transfected with mimic miR-378 relative to control ones. **a**, **c**-**f** Statistical analysis was performed applying *t*-test. ***p < 0.001, *p < 0.05. **g** Western blot analysis of uPAR protein expression in M14 and A375 cells transiently transfected with mimic miR-378 or with miR-378 inhibitor (anti-miR-378) and the relative miRNA scramble control (mimic Ctrl; anti-miR-Ctrl). **b**, **g** Representative images of one out of three independent experiments are reported. HSP72/73 and α-tubulin were used as loading and transferring control.

As observed in other tumor histotypes^13,25^, miR-378a-5p overexpression induced a significant increase of both migratory and invasive capacity of M14, A375, and SBCL1 melanoma cells **(**Fig. 2a, Supplementary Fig. 3), as well as an increased expression of Metalloprotease-2 (MMP2) (Fig. 2b), which is a key metalloprotease involved in melanoma progression.

We further analyzed the involvement of miR-378a-5p in vasculogenic mimicry (VM), an alternative way to provide tumor blood perfusion^26^. As reported in Fig. 2c and Supplementary Fig. 4, the formation of channel-like structures, evaluated in terms of number of intersections, was augmented in M14 and SBCL1 melanoma cells overexpressing miR-378a-5p, compared with control cells.

We next investigated on the possible factors involved in miR-378a-5p-induced VM. VM depends on the expression of several factors including VEGF and interleukin-8 (IL-8) and both these factors are modulated by miR-378a-5p in lung carcinoma cells^27^. Thus, we evaluated whether VEGF and IL-8 are regulated by miR-378a-5p in melanoma models and their impact on miR-378a-5p-induced VM. In agreement with previously reported data^27,28^, miR-378a-5p induced a significant increase of VEGF secretion in M14 and A375 cells (Fig. 2d). Of note, neutralizing antibodies directed versus VEGF were able to strongly reduce miR-378a-5p-induced VM in M14 and SBCL1 cells (Fig. 2e, Supplementary Fig. 4a, b). On the contrary, IL-8 was found to be equally secreted in control and miR-378a-5p overexpressing cells (data not shown), and IL-8-neutralizing antibodies did not affect miR-378a-5p-induced VM (Fig. 2e, Supplementary Fig. 4).

We also evaluated whether miR-378a-5p was able to affect the expression of uPAR a very critical regulator of migration, invasion and VM^29^. The modulation of miR-378a-5p in melanoma cells shows a significant regulation of uPAR expression at transcriptional (Fig. 2f) and protein (Fig. 2g) level.

### miR-378a-5p negatively regulates HOXD10 expression

**Fig. 3:**
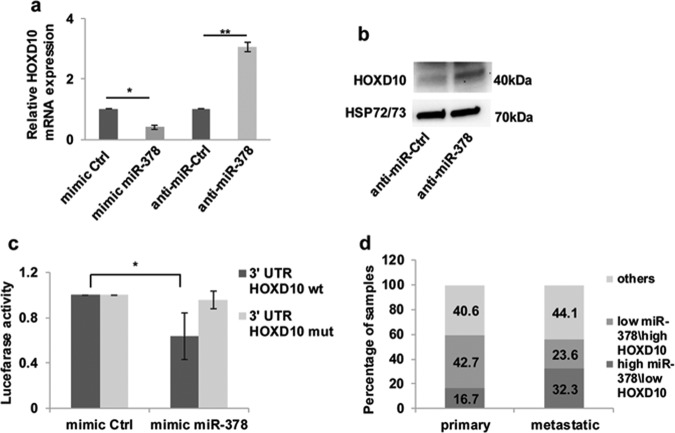
miR-378a-5p (here abbreviated to miR-378) negatively regulates HOXD10 expression in melanoma cells. qRT-PCR analysis of HOXD10 mRNA expression in M14 cells transfected with **a** mimic miRNA scramble control (mimic Ctrl), mimic miR-378 or miR-378 inhibitor (anti-miR-378) and the relative miRNA scramble control (anti-miR-Ctrl). The results are reported as fold induction ± SEM in cells transfected with mimic miR-378 or anti-miR-378 respect to relative controls. **b** Western blot analysis of HOXD10 protein expression in M14 cells transiently transfected with anti-miR-Ctrl or anti-miR-378. Representative images of one out of two independent experiments are reported. HSP72/73 was used as loading and transferring control. **c** Luciferase assay showing miR-378 binding to 3′UTR region of HOXD10 mRNA. Expression vectors carrying a luciferase reporter followed by the 3′-UTR regions of HOXD10 in their *wild-type* (wt, black bars) or mutated (mut, gray bars) forms, in the miR-378 complementary sequence were transfected in A375 cells in the presence of mimic Ctrl or mimic miR-378. Normalized luciferase activities of mimic miR-378 transfected cells respect to control were reported. **a**, **c** Statistical analysis was performed applying *t*-test. *p < 0.05, **p < 0.01. **d** Contingency table showing the distribution of high miR-378/low HOXD10 (*N* = 123, z score > 0) and low miR-378/high HOXD10 (*N* = 119, z score < 0) in primary (*N* = 57) and metastatic (*N* = 185) samples (*p-*value = 0.0001 using Fisher’s exact test). The high or low levels of miR-378 and HOXD10 expression were defined based on positive or negative z-scores of the miRNA\gene expression. In particular, z-score miR-378 > 0 & z-scores gene < 0 for high miR\low HOXD10; z-score miR-378 < 0 & z-score gene > 0 for low miR\high HOXD10.

As miR-378a-5p negatively regulates the expression of SP1 (Supplementary Fig. 1), a transcription factor demonstrated to positively regulate the expression of uPAR^30^, we searched for other possible transcription factors involved in miR-378a-5p-induced uPAR expression. To this purpose we performed qRT-PCR and western blot analyses to evaluate whether miR-378a-5p affects the expression of HOXD10^31^, a transcription factor known for its ability to repress the expression uPAR and some MMPs in cancer^31,32^. As reported in Fig. 3a, b, the modulation of miR-378a-5p affected the expression of HOXD10 both at the transcriptional and protein level, thus indicating that miR-378a-5p may target HOXD10. This hypothesis is supported by bioinformatics analysis predicting HOXD10 as a putative miR-378a-5p target gene (Supplementary Table 1) and confirmed by luciferase assay, showing the binding of miR-378a-5p to the wild-type 3′UTR region of HOXD10 mRNA. As shown in Fig. 3c, by co-transfecting the miR-378a-5p and the plasmid containing the 3′UTR region of HOXD10 mRNA, the signal of luciferase activity was significantly decreased due to the binding of the miRNA to its target sequence. The mutation of the binding sequence in the 3′UTR region of HOXD10 mRNA restored the basal luciferase activity (Fig. 3c). Moreover, the expression profiling analysis of HOXD10 in the same melanoma patient cohort from the TCGA database used to evaluate the expression levels of miR-378a-5p reported in Fig. 1a, showed negative correlation between HOXD10 and miR-378a-5p levels (R = −0.24, *p-*value = 2.7 × 10^–7^) and a lower level of HOXD10 transcript in metastatic melanoma samples compared to primary ones, although with a non-significant *p-*value (0.06) (data not shown). Interestingly, contingency table (Fig. 3d) demonstrated about 32% of high miR-378a-5p/low HOXD10 in metastatic samples while only about 17% was evidenced in primary samples (*p-*value = 0.0001; Pearson’s R = −0.72).

**Fig. 4:**
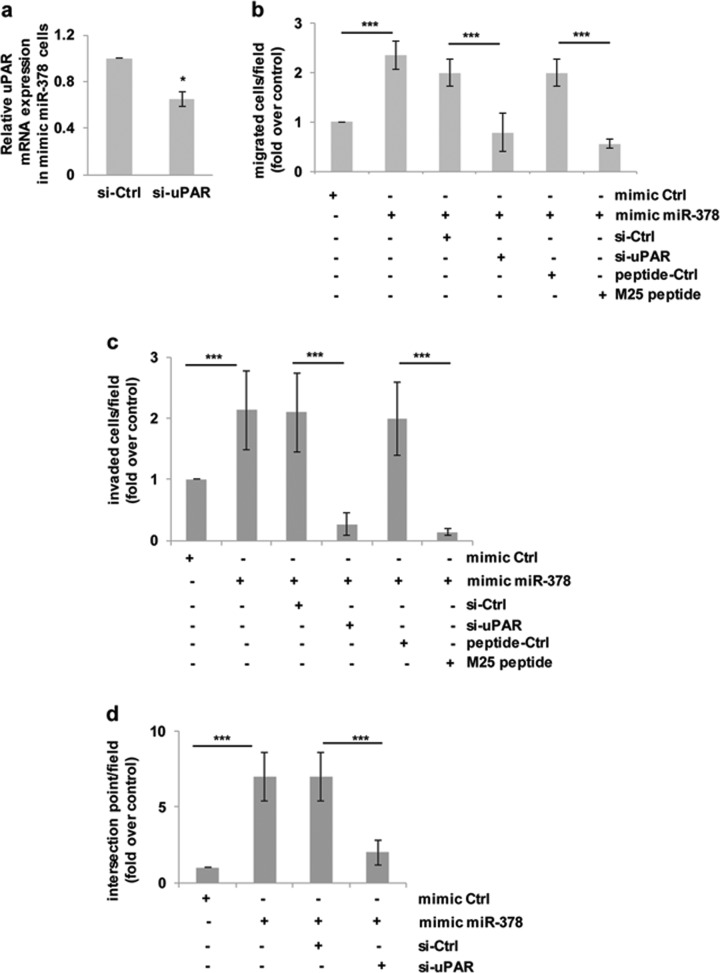
miR-378a-5p (here abbreviated to miR-378) promotes migration, invasion and vasculogenic mimicry of melanoma cells through uPAR. **a** qRT-PCR analysis of uPAR mRNA expression in M14 cells transfected with mimic miR-378 and with siRNA against uPAR (si-uPAR) or the relative scramble control siRNA (si-Ctrl). The results are reported as fold ± SEM in cells transfected with si-uPAR respect to si-Ctrl. In vitro cell migration (**b**) and invasion (**c**) of M14 cells transiently transfected with mimic scramble miRNA control (mimic Ctrl), or mimic miR-378, or mimic miR-378 and siRNA oligonucleotides directed against uPAR (si-uPAR, 20 nM, 48 h), or M25 peptide (50 μM, 2 h) and relative scramble controls (si-Ctrl or peptide Ctrl, respectively). Values are expressed as fold of migrated/invaded cells respect to mimic Ctrl. **d** Evaluation of capillary-like structures formation in M14 cells transfected with mimic Ctrl, or mimic miR-378, or mimic miR-378 and si-uPAR or its relative control si-Ctrl. The fold of intersection points/field respect to mimic control was reported. **a**–**d** Data were expressed as average ± SD. Statistical analysis was performed applying *t*-test. *p < 0.05; ***p < 0.001.

Our data together with the ability of uPA/uPAR axis to function as a degrader of extracellular matrix and a regulator of migration, invasion and VM^30^, are indicative of a possible involvement of uPA/uPAR axis in miR-378a-5p-induced in vitro tumor-promoting functions. To investigate the relevance of uPAR in the ability of miR-378a-5p to affect in vitro properties associated with melanoma aggressiveness, a specific small interference RNA smart pools (si-uPAR) able to reduce uPAR expression^33^ (Fig. 4a) was used after miR-378a-5p mimic transfection. As reported in Fig. 4b–d and Supplementary Fig. 5, 6, uPAR silencing strongly reduced miR-378a-5p ability to increase in vitro cell migration, invasion and VM, when compared to the relative control.

Through its interaction with integrins, we previously reported that uPAR is able to affect melanoma invasion, migration and response to therapy^33^. We also demonstrated that the M25 linear peptide was able to uncouple uPAR from integrins thus affecting its functions^33,34^. On the basis of these evidences, we reasoned that inhibition of uPAR functions with M25 peptide could produce functional effects similar to those obtained with uPAR silencing by means of si-uPAR. As reported in Fig. 4b, c and Supplementary Fig. 5, M25 peptide strongly reduced miR-378a-5p-induced migration and invasion, when compared to cells treated with the relative control peptide.

### miR-378a-5p enhances in vitro and in vivo angiogenesis

**Fig. 5:**
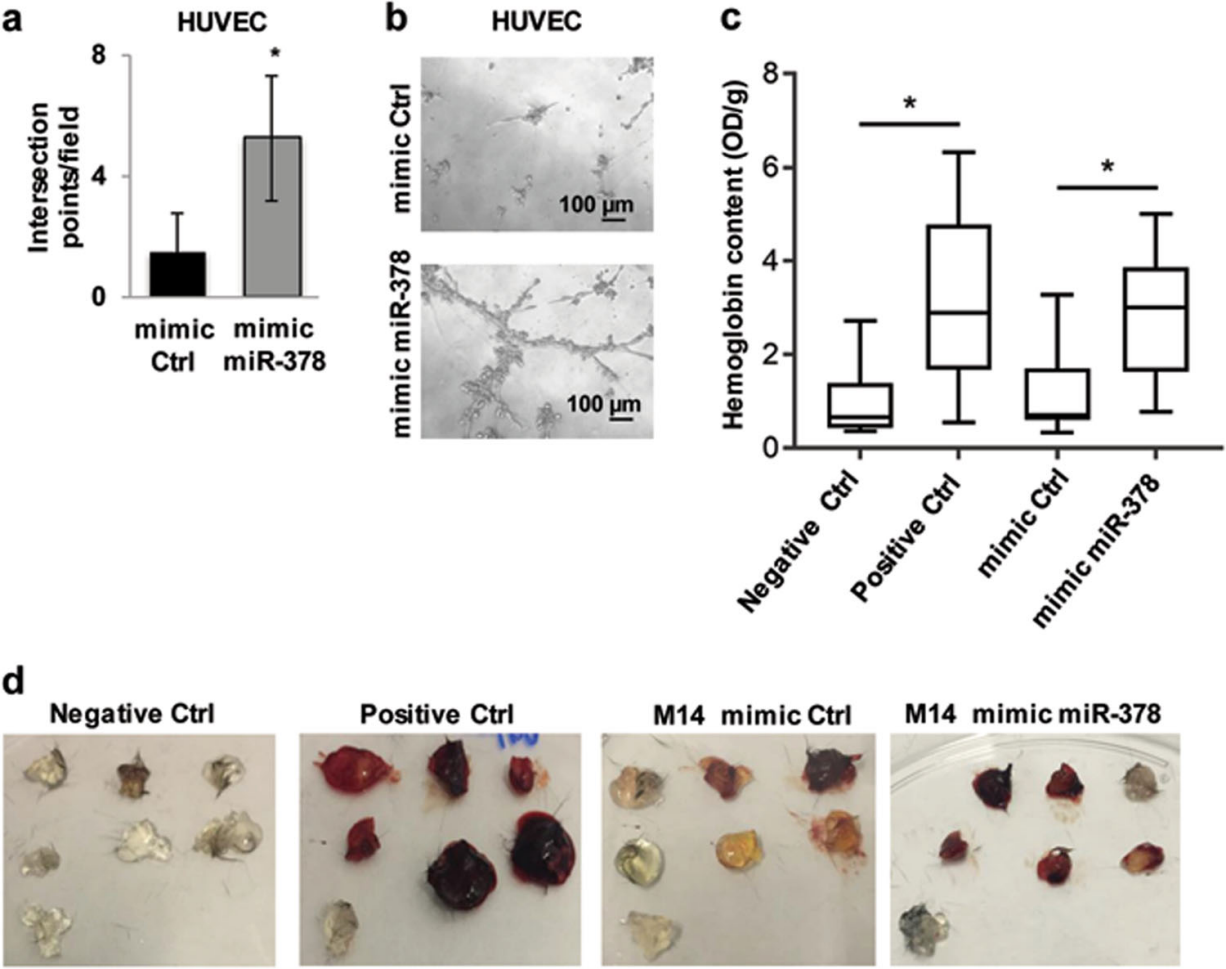
miR-378a-5p (here abbreviated to miR-378) enhances in vitro and in vivo angiogenesis. **a** Endothelial capillary tube-like network formation evaluated in HUVEC seeded on Cultrex BME and exposed to conditioned medium (CM) derived from mimic control (mimic Ctrl) or mimic miR-378 overexpressing M14 cells. The average ± SD of three independent experiments performed in triplicate is reported. Statistical analysis was performed applying *t*-test. *p < 0.05. **b** Representative images of one representative experiment performed as reported in **a**. **c** Box and Whisker plot showing results quantification of the haemoglobin content in the Matrigel plugs containing CM from M14 cells transiently transfected with mimic Ctrl or mimic miR-378. In the negative and positive controls, the CM was replaced with serum-free medium or VEGF, respectively. Statistical analysis was performed applying Mann-Whitney test, *p < 0.05. **d** Images of one representative experiment performed as reported in **c**.

As reported in Fig. 2d, the secretion of the proangiogenic factor, VEGF, was significantly increased in melanoma cells after miR-378a-5p overexpression. Moreover, miR-378a-5p is exported from lung cancer cells in exosomes and the secretion of this miRNA has been reported to correlate with miR-378a-5p expression by the cells^28^. Thus, we investigated whether miR-378a-5p was able to affect the in vitro and in vivo angiogenesis. As shown in Fig. 5a, b, human umbilical endothelial cells (HUVEC) seeded on Cultrex BME and exposed to conditioned medium (CM) derived from M14 cells overexpressing mimic miR-378a-5p, formed a significant increased number of tubular-like structures when compared to cells exposed to CM from control cells. In agreement with the in vitro results, matrigel plugs containing CM from mimic miR-378a-5p overexpressing M14 cells injected in C57Bl6 mice, showed a higher co-option of surrounding vessels and about 4-fold induction of the haemoglobin content when compared to the matrigel plugs containing CM from control cells (Fig. 5c, d).

### Bcl-2 modulation affects miR-378a-5p expression

**Fig. 6:**
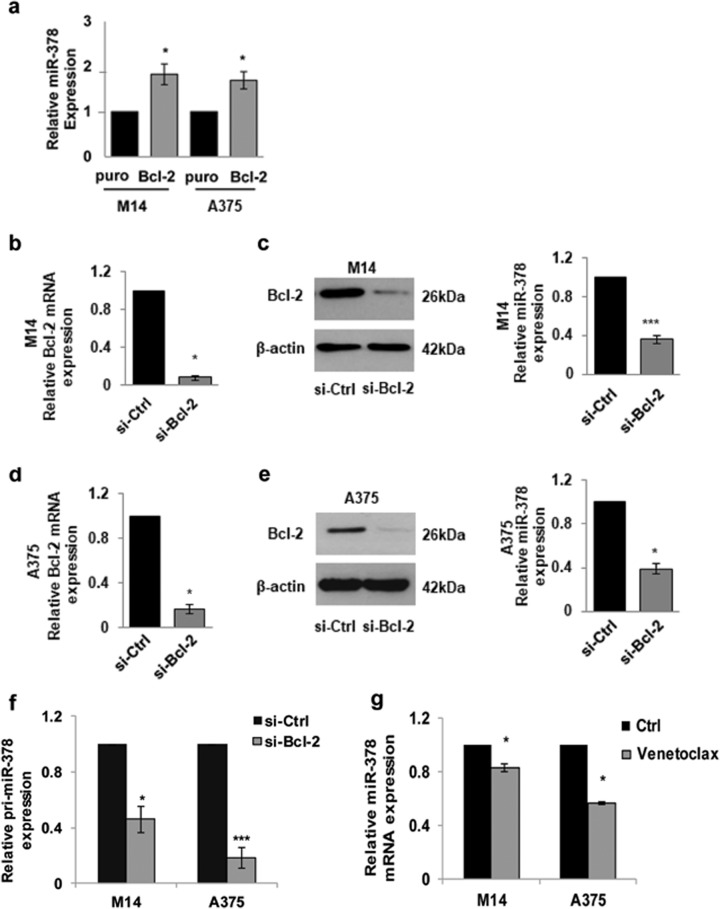
Bcl-2 protein modulates miR-378a-5p (here abbreviated to miR-378) expression in melanoma cells. qRT-PCR analysis of miR-378 expression in **a** M14 and A375 control (puro) and Bcl-2 overexpressing (Bcl-2) melanoma cells and in **b** M14 and **d** A375 cells transfected with siRNA oligonucleotides directed against Bcl-2 (si-Bcl-2) or scramble control siRNA (si-Ctrl). **c**, **e** Western blot analysis and qRT-PCR of Bcl-2 protein and mRNA levels in **c** M14 and **e** A375 cells transfected with si-Bcl-2 or si-Ctrl. Representative images of one out of two independent western blotting experiments are reported. β-actin was used as loading and transferring control. **f** qRT-PCR analysis of pri-miR-378a-5p (pri-miR-378) expression in M14 and A375 cells transfected with si-Bcl-2 or si-Ctrl. **g** qRT-PCR analysis of miR-378 expression in M14 and A375 cells treated with Venetoclax, a specific Bcl-2 inhibitor (5 μM for 72 h). **a**, **b**, **d**, **f**, **g** The average ± SEM of three independent experiments performed in triplicate is reported. Statistical analysis was performed applying *t*-test, *p < 0.05, ***p < 0.001.

We previously reported Bcl-2 ability to regulate the expression of miR-211 and miR-204, two miRNA involved in melanoma progression and resistance^2^. To evaluate whether the expression of miR-378a-5p was modulated by Bcl-2, we used either Bcl-2 overexpressing clones previously obtained and characterized^2^, or melanoma cells in which Bcl-2 expression was silenced by siRNA^2^. Increased miR-378a-5p expression was found in M14 and A375 cells overexpressing Bcl-2 (Fig. 6a), while a reduced expression of miR-378a-5p was observed after Bcl-2 silencing both in M14 (Fig. 6b, c) and A375 (Fig. 6d, e) cells. Bcl-2 downregulation with siRNA also decreased pri-miR-378a-5p (pri-miR-378) expression (Fig. 6f). Interestingly, treatment with Venetoclax, a specific Bcl-2 inhibitor^35^, significantly reduced miR-378a-5p expression in melanoma cells (Fig. 6g).

## Discussion

Motivated by the lack of published results on the relevance of miR-378a-5p in melanoma malignancy, in this study we explored the biological and functional role of this miRNA by using melanoma preclinical models and human melanoma specimens.

Our results originally demonstrated, that miR-378a-5p acts as an oncogenic miRNA in melanoma. In particular, evaluating the expression levels of miR-378a-5p in a large melanoma patient cohort from TCGA database we found a significantly upregulated expression in metastatic melanoma when compared to primary ones. A significant upregulation of miR-378a-5p was also observed in malignant melanoma specimens when compared to melanoma in situ obtained from our Institute, and in melanoma cells resistant to BRAF or MEK inhibitors compared to sensitive ones. Interestingly, treatment with Trametinib, a MEK inhibitor, significantly reduced the expression of miR-378a-5p, thus indicating that miR-378a-5p is affected by MEK signaling.

Contrary to the results demonstrating miR-378a-5p ability to affect proliferation of several tumor histotypes^14,19,23^, we did not observe any effect of miR-378a-5p either on proliferation or clonogenic ability of melanoma cells, thus indicating a cell-type specific regulation. On the contrary, we demonstrated miR-378a-5p ability to increase in vitro cell migration, invasion, and VM. The latter represents an alternative way to provide sufficient blood perfusion through the formation of embryonic vasculogenic networks that is necessary for growth and metastatization of several tumors, including melanoma, and is strongly related to poor clinical outcome of melanoma patients^36^. We also demonstrated miR-378a-5p-dependent regulation, at both transcriptional and protein level, of uPAR, a proteolytic enzyme that degrades extracellular matrix, is strongly associated with invasion and metastasis in melanoma^37^, contributes to VM^38^, and supports endothelial cell proliferation, migration and invasion^39^. Genetic (si-uPAR) and pharmacologic (M25 inhibitor peptide) approaches also demonstrated the relevance of uPAR in the tumor-promoting functions of miR-378a-5p.

In agreement with previously published papers^14,19,23^ our results confirmed miR-378a-5p ability to negatively regulate the expression of KLF9, FUS-1, and SUFU, transcription factors with tumor suppression function, involved in cell survival, growth, and angiogenesis of several cancer models not including melanoma^14,15,23,25^. We also identified STAMBP and HOXD10 as new miR-378a-5p target genes, being miR-378a-5p able to bind the 3′UTR region of both STAMBP and HOXD10 mRNA. We focused our attention on HOXD10, a previously reported transcriptional repressor of uPAR and some MMP in cancer^31,32^, and identified by in silico analysis as a putative miR-378a-5p target. We demonstrated that miR-378a-5p inhibition increased HOXD10 mRNA expression while miR-378a-5p overexpression was able to negatively affect HOXD10 level. HOXD10 relevance in melanoma progression and its involvement in miR-378a-5p mediated functions is strongly supported by: (i) the lower level of HOXD10 transcript in metastatic melanoma samples compared to primary ones observed in a TCGA dataset, (ii) the negative correlation between miR-378a-5p and HOXD10 expression levels observed in a TCGA dataset of a melanoma patient cohort, (iii) the role played by HOXD10 in the regulation of MMP2^40^ and in maintaining a non-angiogenic state in the endothelium^31^.

We cannot exclude that miR-378a-5p might also indirectly exert its effect on uPAR expression through the modulation of other factors, such as co-factors/proteins reported to enhance its mRNA stability (i.e., HUR, hnRNPC, miR10b, and miR23-a)^41^, or transcription factors, such as SOX2, that is significantly (*p-*value = 0.0007) induced in our experimental models (data not shown) and is a predicted transcription factor for uPAR by bioinformatic analysis (*p-*value = 0.00065; https://biogrid-lasagna.engr.uconn.edu/). The association observed between miR-378a-5p and KLF9 expression in the present study also raises the possibility that the latter could influence the invasive behaviour of melanoma cells through its effect on uPAR, VEGF or MMP^42^.

We also evidenced a proangiogenic property of miR-378a-5p in melanoma cells. In agreement with previously reported data^27,28^, miR-378a-5p induced a significant increase of VEGF secretion in melanoma cells while, contrary to the evidence that miR-378a-5p increases the expression of IL-8^28^, the cytokine was found to be equally secreted in control and miR-378a-5p overexpressing melanoma cells, thus indicating a cell-type specific effect. Accordingly, by using endothelial cells and in vivo matrigel assay we evidenced a proangiogenic property of miR-378a-5p in melanoma cells. In agreement with our results, tumors formed by miR-378a-5p-transfected glioblastoma and lung carcinoma cells contain, respectively, larger blood vessels^14^ or better-vascularized tumors^28^.

Collectively, this study and a previous one^2^, also indicate that Bcl-2 is a regulator of miRNA biogenesis and function, and that it could affect in vitro melanoma progression-associated properties through its effect on miRNA expression. Moreover, treatment with a specific inhibitor of Bcl-2 induced downregulation of miR-378a-5p in melanoma cells, thus indicating that pharmacologic inhibition of Bcl-2 could exert its effect also through modulation of miR-378a-5p functions.

In conclusion, we showed that the oncogenic activity of miR-378a-5p in melanoma functions includes two regulatory events: an increase of in vitro cell invasion, migration and VM through HOXD10/uPAR axis, and an increase of in vitro and in vivo angiogenesis through VEGF. The experimental evidences are supported by an analysis of melanoma clinical samples in which miR-378a-5p expression was higher in melanoma metastasis than in primary tumors.

The salient finding of the present study is the demonstration that miR-378a-5p acts as a positive regulator of in vitro melanoma progression-associated properties. The scheme reported in Supplementary Fig. 7 depicts: (a) STAMBP and HOXD10 as new miR-378a-5p target genes, in addition to the previously reported ones^14,16,20,23,43–49^; (b) miR-378a-5p regulation by Bcl-2 identified beyond the already demonstrated players affecting miR-378a-5p expression^28,43,50^ and biologic effects observed in melanoma. This study sheds light on the possibility of targeting oncogenic mi-R378a-5p for melanoma therapy. It is possible to envision that targeting this miRNA might be useful to inhibit cancer invasion by preventing the upregulation of uPAR, and tumor angiogenesis by preventing the upregulation of VEGF. Since the activity of miR-378a-5p on cell growth is controversial and is related to the cellular context, careful analysis of miR-378a-5p targeted genes should be performed to determine the pathophysiological properties of melanoma.

## Materials and methods

### Patient tissue samples

RNA from formalin-fixed paraffin-embedded metastatic (27 cases) and in situ (13 cases) melanoma specimens collected at Istituti Fisioterapici Ospitalieri^51^ were extracted using miRNeasy FFPE kit (Quiagen, Hilden, Germany) and retrospectively used for qRT-PCR analysis of miR-378a-5p expression. The ethics committee of the Regina Elena National Cancer Institute (Prot. CE/913/10) approved the use of human tissue samples according to biobanca criteria. Informed consent was obtained from all subjects.

### Bioinformatic analysis of TCGA dataset

We used a melanoma patient cohort, including 351 metastatic and 96 primary melanoma specimens, from TCGA (http://cancergenome.nih.gov/). miR-378a-5p and miR-378a-3p deregulation was assessed by applying Mann-Whitney test. Significance was defined at the *p* < 0.05 level.

### In silico miRNA targets identification

Prediction target tools were interrogated by using the web server tool MirWalk3 (http://mirwalk.umm.uni-heidelberg.de/)^52^.

### Cell lines

M14 and A375 human melanoma cell lines were purchased from American Type Culture Collection (Manassas, VA). SBCL1 human melanoma cell line was provided by Bruno Giovanella^53^. A375 human melanoma cells sensitive (A375S), resistant to Vemurafenib (A375R) or to Dabrafenib and Trametinib (A375DR), were previously characterized^22^. HUVEC (PromoCell GmbH, Heidelberg, Germany) were cultured in complete EBM-2 medium (Clonetics Bio Whittaker, now Lonza, Cologne, Germany), containing 2% fetal bovine serum (FBS). All cell lines were grown in RPMI medium (Euroclone, Milan, IT) supplemented with 10% (v/v) FBS, 1% penicillin/streptomycin and 1% L-glutamine (Euroclone) at 37 °C in a balanced air humidified incubator with 5% CO_2._ They have been routinely tested for mycoplasma contamination and were recently authenticated (STR profiling) and tested for mycoplasma contamination.

### Plasmids and transfections

For mature miR-378a-5p expression, we used mirVana™ miRNA Mimic Negative Control #1 or hsa-miR-378-5p mirVana™ miRNA Mimic (Ambion Inc., Austin, Texas, USA). For miR-378a-5p depletion, we used mirVana™ miRNA Inhibitor Negative Control #1 or hsa-miR-378a-5p mirVana™ miRNA Inhibitor (Ambion Inc.). For miRNA transfection, cells were seeded and after 24 h transfected at final concentration of 5 nM for miRNA mimics for 72 h, and at final concentration of 10 nM for miRNA inhibitors for 24 h, by using INTERFERin^®^ (Polyplus Co., Sébastien Brant Illkirch, France) according to the manufacturer’s instructions. After transfection, miR-378a-5p expression was assessed by qRT-PCR.

For stable Bcl-2 transfections, melanoma cell lines were transfected using Lipofectamine (Invitrogen, Carlsbad, CA) as previously reported^54^, and clones cultured in the presence of 1 μg/ml puromicine (Sigma-Aldrich, St Louis MO).

Pooled siRNA oligonucleotides against uPAR (si-uPAR) (33), Bcl-2 (si-Bcl2)^2^, or scramble (si-Ctrl) target sequences were purchased from Dharmacon RNA Technologies (siGENOME SMART pool, DharmaconRNA Technologies, Lafayette, CO, USA). For siRNA transfection, cells were seeded and after 24 h transfected with 20 nM pooled oligonucleotides mixture by using jetPRIME (Polyplus Co.) following the manufacturer’s protocol. After 24 h, the medium was changed and uPAR or Bcl-2 mRNA expression were assessed 48 h after silencing by qRT-PCR and western blotting.

### Treatment of cells with M25 peptide

Inhibition of uPAR-integrin interaction was obtained with the M25 peptide (STYHHLSLGYMYTLN), previously identified in a phage display library^55^ and produced by PRIMM srl (Milan, Italy). Its sequence spans an exposed loop on the ligand-binding surface of integrin α chain and uncouples the interaction of the α-chain of integrin with uPAR, thus impairing uPAR functions^33,34^. Fifty μM M25 and control scramble peptide were used for in vitro experiments (2 h for migration and invasion).

### Lysate preparation and immunoblotting analysis

Cells were lysed in 10 mM Tris-HCl buffer (pH 7,4) with 2% SDS and fresh protease inhibitors. Extracts were sonicated for 20 sec and protein concentrations were determined by colorimetric assay (Pierce™ BCA Protein Assay Kit, Thermo Scientific, Waltham, Massachusetts, USA). Western blotting was performed using the following primary antibodies: α-tubulin (DM1A, sc- 32293), Bcl-2 (100, sc-509), Homeobox D10 (HOXD10) (G-3, sc-166235), KLF9 (A-5, sc-376422), MMP2 (H-76, sc-10736), SP1 (1C6, sc-420), STAMBP (H-4, sc-271641), SUFU (F-4, sc-137014), uPAR (FL-290, sc-10815, Santa Cruz Biotechnology) from Santa Cruz Biotechnology (Santa Cruz, CA), β-actin from Sigma-Aldrich and HSP72/73 (Ab-1; HSP-01) from Calbiochem (Germany). Secondary antibodies used were anti-mouse and anti-rabbit, conjugated to horseradish peroxidase (Amersham Biosciences, Piscataway, NJ). Immunostained bands were detected by chemiluminescent method (Pierce, Thermo Scientific). At least two independent experiments were performed for each protein detection.

### ELISA

The level of secreted VEGF and IL-8 by melanoma cells was assayed by ELISA kit according to the manufacturer’s instructions (R&D Systems, Minneapolis, MN, USA) normalising the supernatants to the number of adherent cells.

### Total RNA extraction and qRT-PCR

Total RNA was extracted using NORGEN Total RNA Purification Plus Kit (Norgen, ON, Canada, USA) following the manufacturer’s instructions. One microgram of total RNA was reverse-transcribed using RevertAid Reverse Transcriptase (Thermo Scientific). qRT-PCR was performed using the SYBR green dye detection method. The mRNA levels were normalized using glyceraldehyde 3-phosphate dehydrogenase or beta-actin. For pri-miRNA and mature miRNA expressions, small amount of RNA (50 ng) was reverse-transcribed using the TaqMan microRNA Reverse Transcription Kit (Applied Biosystem) and real-time-PCR was carried out in a final volume of 20 μl using a 7900HT Fast Real-Time PCR System (Applied Biosystems). The PCR Reactions were initiated with a 10 min incubation at 95 °C followed by 40 cycles of 95 °C for 15 sec and 60 °C for 60 sec. qRT-PCR was performed using TaqMan MicroRNA Assays (Applied Biosystems) according to the manufacturer’s protocol. RNU19 was used as endogenous control to normalize miRNA expression. Values are reported as fold changes compared to melanoma control cells using the 2^–ΔΔCt^ method. Primers used are listed in Supplementary Table 2.

### Luciferase reporter assay

The OriGene (OriGene Technologies GmbH, Herford, Germany) 3′UTR- luciferase reporter vector MirTarget SC208375 was used to study the interaction between STAMBP and miR-378a-5p. The 3′UTR sequence of the human STAMBP gene was inserted downstream of the luciferase gene into the dual luciferase reporter plasmid.

The pMIR-REPORT Luciferase miRNA expression reporter vector containing the 3′UTR sequence of the human HOXD10 gene inserted downstream of the luciferase gene, pMIR-D10 UTR, was a gift from Bob Weinberg (Addgene plasmid #19117)^25^.

STAMBP and HOXD10 3′UTR mutant were made with the QuikChange II site-directed mutagenesis kit (Agilent Technologies, Santa Clara, CA, USA), using the primers reported in Supplementary Table 2, and sequenced to confirm the mutated product. Cells were transfected using jetPRIME^®^ (Polyplus Co.) with 200 ng of the plasmid containing the 3′UTR putative target gene, wild-type or mutated, together with 10 nM of mimic Control or mimic miR-378 in 24-well plates. Firefly and Renilla luciferase activities were measured 48 h post transfection using the Dual Luciferase Reporter Assay System in the GloMax 96 Microplate Luminometer (Promega). Firefly luciferase activity of each sample was normalized to Renilla luciferase activity and expressed as and fold activation relative to the basal activity of empty vector.

### Migration and invasion assay

For migration assay, transfected 5 × 10^4^ cells were seeded in serum-free media into the upper chamber of Transwell (Corning, Costar, New York, USA). The lower well contained medium with 10% FBS. For invasion assay, transfected 7.5 × 10^4^ cells were seeded in serum-free media into the upper chamber of commercially available inserts having a polycarbonate membrane coated with a thin basement membrane (CultreCoat^®^ 24-Well Medium BME Cell Invasion Inserts, Trevigen, Gaithersburg, MD, USA). After 18 h incubation at 37 °C, cells remaining on the top side of the membrane were removed, migrating and invading cells fixed, stained, photographed, and counted. The experiment was performed in triplicates for all conditions described. From each transwell, several images were taken under a phase-contrast microscope at ×4 magnification and two broad fields were considered for quantification.

### Analysis of vasculogenic mimicry

Two hundred and fifty microliters of polymerized Cultrex BME (12–18 mg/ml, Trevigen) were dropped onto each well of a 24-well plate and were allowed to solidify for 1 h at 37 °C in humidified 5% CO_2_ incubator. Transfected 2 × 10^5^ melanoma cells were seeded in serum-free medium onto the gelled BME and incubated at 37 °C for 18 h. Cells were incubated alone or in the presence of specific human IL-8- or VEGF-neutralizing antibodies (0.2 μg/mL, CXCL8-MAB208 and VEGF-MAB293, R&D Systems) and capillary-like structures (CLS) evaluated. CLS formation was photographed using light microscopy and quantified by evaluating the tube length and counting the number of cell junctions in 10 sets of images for condition. Each condition was analyzed in duplicate in three different experiments using image analysis program (Image J v.1.34 s; http://rsb.info.nih.gov/ij/).

### In vitro morphogenesis assay

Two hundred and fifty microliters of polymerized Cultrex BME (12–18 mg/ml) were added to each well of precooled 24-well tissue culture plate. Pipette tips and BME solution were kept cold throughout to avoid solidification. The plate was incubated at 37 °C for 1 h to allow the matrix solution to solidify. A total of 2 × 10^5^ HUVEC, were seeded on BME and exposed to CM derived from control or mimic miR-378a-5p overexpressing cells. After 8 h, capillary tube-like network formation (morphogenesis) was observed as reported for melanoma cells.

### In vivo matrigel assay

In vivo Matrigel assay and quantification of the hemoglobin content in the Matrigel plugs were done as previously reported^54^ using CM from 5 × 10^6^ viable cells after transfection with control or mimic miR-378a-5p. Groups of eight female mice were used for each experimental point. In the negative and positive controls, the CM was replaced with serum-free medium or with VEGF, respectively. We created the different groups of mice without any specific randomization scheme.

All procedures involving animals and their care were conducted in conformity with the institutional guidelines, which are in compliance with national and international laws.

### Statistical analysis

All results are expressed as mean ± SEM or standard deviation (SD) (as specified in the Figures legends) of at least three independent experiments performed in triplicate. All qRT-PCR experiments were carried out in triplicate. Differences between groups were analyzed with a two-sided paired or unpaired *t*-test or Mann-Whitney Test when appropriated and were considered statistically significant for *p* < 0.05. The sample size has been chosen based on our previous experience.

## Supplementary information

Table S1

Table S2

Supplementary Figure Legends

Supplementary Figures
