# Supplementary material for: microRNA-378a-5p iS a novel positive regulator of melanoma progression
Source: Oncogenesis. 2020 Feb 14;9(2):22. doi: 10.1038/s41389-020-0203-6 (PMC7021836; doi:10.1038/s41389-020-0203-6)
Supplement: Supplementary file 1 — Table S1 [file 41389_2020_203_MOESM1_ESM.docx]

Table S1. List of mir-378a-5p target genes

| gene | Ref Seq ID | Score*(miRWalk**) | position | validation |
| --- | --- | --- | --- | --- |
| KLF9 | NM_001206 | - | 3’-UTR | Peng N et al 2018 |
| SUFU | NM_016169 | - | 3’-UTR | Lee DY et al 2007 |
| FUS-1/TUSC2 | NM_004960 | 1.0 | 3’-UTR | Lee DY et al 2007 |
| SP1 | NM_138473 | 1.0 | 3’-UTR | - |
| HOXD10 | NM_002148 | 0.923 | 3’-UTR | - |
| STAMBP | NM_201647 | 0.846 | 3’-UTR | - |

* The miRWalK Score is calculated from a random-forest based approach by executing TarPmiR algorithm for miRNA target site prediction. Based on the training data, it shows the probability that this interaction "works". The random-forest-based approach software TarPmiR searching the complete transcript sequence including the 5’-UTR, CDS and 3’-UTR

**http://mirwalk.umm.uni-heidelberg.de/ (Sticht C, De La Torre C, Parveen A, Gretz N. miRWalk: An online resource for prediction of microRNA binding sites. PLoS One 2018;13: e0206239; Dweep, H et al. “miRWalk2.0: a comprehensive atlas of microRNA-target interactions” Nature Methods, 12(8): 697-697 (2015)).
