# Supplementary material for: microRNA-378a-5p iS a novel positive regulator of melanoma progression
Source: Oncogenesis. 2020 Feb 14;9(2):22. doi: 10.1038/s41389-020-0203-6 (PMC7021836; doi:10.1038/s41389-020-0203-6)
Supplement: Supplementary file 2 — Table S2 [file 41389_2020_203_MOESM2_ESM.docx]

Table S2: List of primers used in qRT-PCR

| List of Primers | **Forward 5'-3'** | **Reverse 5'-3'** |
| --- | --- | --- |
| Bcl-2 | CTGCACCTGACGCCCTTCACC | CACATGACCCCACCGAACTCAAAGA |
| FUS-1 | AGCGGTGTTGGAACTTCGTT | CGTGGACTGGCTATAACCACTG |
| GAPDH | TCCCTGAGCTGAACGGGAAG | GGAGGAGTGGGTGTCGCTGT |
| KLF9 | GGCTGTGGGAAAGTCTATGGAA | GCGGGAGAACTTTTTAAGGCAG |
| SP1 | AAACGTTTCACACGTTCGGA | TGCATGACGTTGATGCCACT |
| STAMBP | GAGATTTCTTCCTGTCGCCAGA | GAAGGTGGACTCAAACGCT |
| SUFU | ATTCCCCATGAGCTGATTCGC | TAAAGTGCCGTCCATGCAGGA |
| uPAR | ATCACCAGCCTTACCGAGGTT | ATGCATTCGAGGTAACGGC |
| HOXD10 | CTCCACTGTCATGCTCCAGCTCAAC | CTTTCTGCCACTCTTTGCAGTGAGCC |
| STAMBP  mut | CCTGAAATATTTATTAAATAACAATAATCAAAATCATTCTTCGGGATGTGTTAGC | GCTAACACATCCCGAAGAATGATTTTGATTATTGTTATTTAATAAATATTTCAGG |
| HOXD10  mut | GTGCCCCCTTCCTGGCCTCTGACCGG | CCGGTCAGAGGCCAGGAAGGGGGCAC |
