# Supplementary material for: microRNA-378a-5p iS a novel positive regulator of melanoma progression
Source: Oncogenesis. 2020 Feb 14;9(2):22. doi: 10.1038/s41389-020-0203-6 (PMC7021836; doi:10.1038/s41389-020-0203-6)
Supplement: Supplementary file 3 — Supplementary Figure Legends [file 41389_2020_203_MOESM3_ESM.pdf]

microRNA-378a-5p IS A NOVEL POSITIVE REGULATOR OF MELANOMA PROGRESSION

Maria Grazia Tupone[1*°], Simona D'Aguanno[1*], Marta Di Martile[1], Elisabetta Valentini[1], Marianna Desideri[1§], Daniela Trisciuoglio[1,2], Sara Donzelli[3], Andrea Sacconi[3], Simonetta Buglioni[4], Cristiana Ercolani[4], Alessio Biagioni[5], Gabriella Fibbi[5], Luigi Fattore[6], Rita Mancini[7], Gennaro Ciliberto[8], Giovanni Blandino[3], Donatella Del Bufalo[1#]

[1]Preclinical Models and New Therapeutic Agents Unit, [3]Oncogenomics and Epigenetics Unit, [4]Pathology Unit and [8]Scientific Direction, IRCCS Regina Elena National Cancer Institute, Rome, Italy; [2]Institute of Molecular Biology and Pathology, National Research Council, Rome, Italy; [5]Department of Experimental and Clinical Biomedical Sciences "Mario Serio", University of Florence, Italy; [6]Laboratory affiliated to Istituto Pasteur Italia, Fondazione Cenci Bolognetti, Department of Molecular and Clinical Medicine, "Sapienza" University and [7]Department of Molecular and Clinical Medicine, "Sapienza" University, Rome, Italy.

#Corresponding author: Donatella Del Bufalo, Preclinical Models and New Therapeutic Agents Unit, IRCCS Regina Elena National Cancer Institute, Via Chianesi 53 (00144) Rome Italy, email: donatella.delbufalo@ifo.gov.it, tel: 003952662575, fax: 00390652662013.

*These authors equally contributed as first authors

°Current address: Department of Life, Health and Environmental Sciences, University of L'Aquila, 67100 L'Aquila, Italy. mariagrazia.tupone@gmail.com.

Running title: microRNA-378a-5p affects melanoma progression

[§]This paper is dedicated to the memory of our wonderful colleague, Marianna Desideri, who made a contribution to this project before she passed away. It is a tribute to her enthusiasm and joyful approach to life and work, and to her friendship.

SUPPLEMENTARY FIGURE LEGENDS

Figure S1: **miR-378a-5p (here abbreviated to miR-378) expression affects the expression of target genes in M14 melanoma cells**. **(a)** qRT-PCR analysis of STAMBP, SP1, KLF9, FUS-1 and SUFU mRNA expression in M14 cells transiently transfected with mimic miR-378a-5p (mimic miR-378) or mimic miRNA scramble control (mimic Ctrl). The results are reported as fold of reduction in mimic miR-378a-5p (mimic miR-378) transfected cells relative to control ones. Putative target prediction was conducted by miRWalk* (http://mirwalk.umm.uni-heidelberg.de/search_mirnas/). The primers used to analyze each gene are listed in Supplementary Table S2. **(b)** Western blot analysis of STAMBP, SP1, KLF9 and SUFU protein expression in M14 cells transfected with mimic Ctrl or mimic miR-378. Representative images of one out of two independent experiments are reported. HSP72/73 was used as loading and transferring control. **(c)** qRT-PCR analysis of STAMBP, SP1, KLF9, FUS-1 and SUFU mRNA expression in M14 cells transiently transfected with miR-378a-5p inhibitor (anti-miR-378) or with mimic miRNA scramble inhibitor control (anti-miR-Ctrl). The results are reported as fold induction in anti-miR-378 transfected cells relative to control ones. **(a,c)** The average ± SEM of three independent experiments performed in triplicate is reported. Statistical analysis was performed applying T-test, **p<0.05, **p<0.01, ***p<0.001*. **(d)** Luciferase assay showing miR-378a-5p binding to 3'UTR region of STAMBP mRNA. Expression vectors carrying a luciferase reporter followed by the 3'UTR regions of STAMBP in their wild-type (wt, black bars) or mutated (mut, gray bars) forms, in the miR-378 complementary sequence were transfected in A375 cells in the presence of mimic Ctrl or mimic miR-378. Normalized luciferase activities of mimic miR-378 transfected cells respect to control were reported. **(a,c,d)** Statistical analysis was performed applying T-test, **p<0.05*.

Figure S2. miR-378a-5p (here abbreviated to miR-378) expression does not affect *in vitro* cell proliferation and colony formation ability of M14 cells. Evaluation of **(a)** *in vitro* proliferation and **(b)** colony formation ability of M14 cells transfected with mimic miR-378a-5p (mimic miR-378), or miR-378a-5p inhibitor (anti-miR-378) or with scramble controls (mimic Ctrl, anti-miR-Ctrl).

Figure S3: miR-378a-5p (here abbreviated to miR-378) expression increases *in vitro* migration and invasion of melanoma cells. Representative images of *in vitro* cell **(a)** migration of M14, A375 and SBCL1 cells and **(b)** invasion of M14 and A375 cells transiently transfected with mimic miRNA scramble control (mimic Ctrl) or mimic miR-378a-5p (mimic miR-378). For *in vitro* cell migration of SBCL1 cells, the quantification of migrated cells/field is reported in the bar chart as

fold respect to cells transfected with mimic control. Statistical analysis was performed applying T-test, ****p<0.001*.

Figure S4: **miR-378a-5p (here abbreviated to miR-378) expression increases *in vitro* vasculogenic mimicry of melanoma cells with a VEGF-dependent mechanism.** Representative images of *in vitro* capillary-like structure formation of (**a**) M14 and (**b**) SBCL1 cells transiently transfected with mimic miRNA scramble control (mimic Ctrl) or with mimic miR-378a-5p (mimic miR378) in absence or presence of VEGF or IL-8 (anti-VEGF or anti-IL-8) neutralizing antibodies. For *in vitro* vasculogenic mimicry of SBCL1 cells, the quantification of intersection points/field is reported in the bar chart. Statistical analysis was performed applying T-test, ****p<0.001*.

Figure S5: **miR-378a-5p (here abbreviated to miR-378) expression increases *in vitro* cell migration and invasion of melanoma cells with a uPAR-dependent mechanism.**

Representative images of *in vitro* cell **(a)** migration and **(b)** invasion of M14 cells transiently transfected with mimic miR-378a-5p (mimic miR-378) incubated with oligonucleotides directed against uPAR (si-uPAR), or with M25 peptide. Scramble siRNA (si-Ctrl) and scramble pepride (peptide-Ctrl) were used as control.

Figure S6: **miR-378a-5p (here abbreviated to miR-378) expression increases *in vitro* vasculogenic mimicry of melanoma cells with a uPAR-dependent mechanism.** Representative images of *in vitro* capillary-like structure formation of M14 cells transiently transfected with mimic miRNA scramble control (mimic miR-Ctrl) or mimic miR-378a-5p (mimic miR-378) incubated with oligonucleotides directed against uPAR (si-uPAR) or scramble siRNA (si-Ctrl).

Figure S7. Schematic figure depicting the identified players in regulating miR-378a-5p expression and its target genes reported in cancer and biological effects observed in melanoma. For target genes, different colors indicate the level of evidence. Blue: changes in mRNA and protein expression after miRNA transfection; Red: changes in mRNA and protein expression after miRNA transfection and proof of direct interaction by reporter assay and site-specific mutagenesis; Green: changes in mRNA and protein expression after miRNA transfection, proof of direct interaction by reporter assay and site-specific mutagenesis and negative correlation of expression in patients. The players and the target genes investigated in the present paper are indicated in bold type.
