# Supplementary material for: microRNA-378a-5p iS a novel positive regulator of melanoma progression
Source: Oncogenesis. 2020 Feb 14;9(2):22. doi: 10.1038/s41389-020-0203-6 (PMC7021836; doi:10.1038/s41389-020-0203-6)
Supplement: Supplementary file 4 — Supplementary Figures [file 41389_2020_203_MOESM4_ESM.pdf]

Figure S1

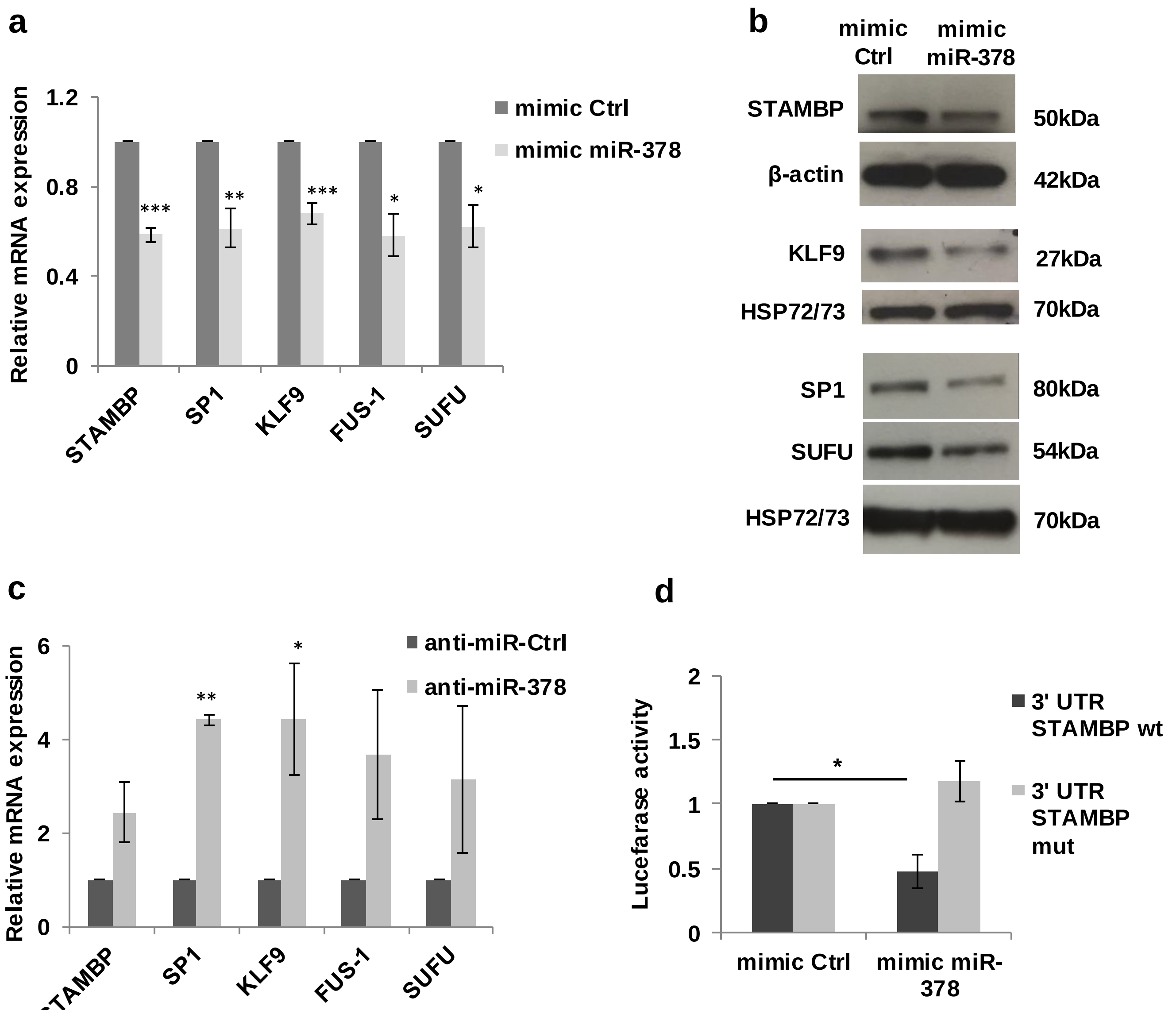
a
Relative mRNA expression
1.2
0.8
0.4
0
mimic Ctrl
mimic miR-378
***
**
***
*
*
STAMBP
SP1
KLF9
FUS-1
SUFU
b
mimic Ctrl
mimic miR-378
STAMBP
50kDa
β-actin
42kDa
KLF9
27kDa
HSP72/73
70kDa
SP1
80kDa
SUFU
54kDa
HSP72/73
70kDa
c
Relative mRNA expression
6
4
2
0
anti-miR-Ctrl
anti-miR-378
**
*
STAMBP
SP1
KLF9
FUS-1
SUFU
d
Lucefarase activity
2
1.5
1
0.5
0
*
3' UTR STAMBP wt
3' UTR STAMBP mut
mimic Ctrl
mimic miR-378

Figure S2

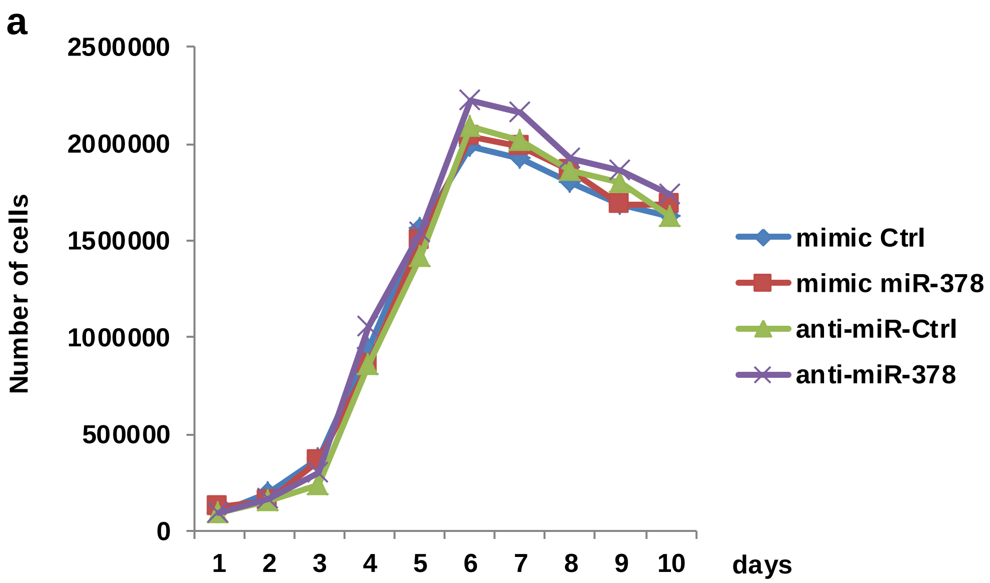
a
2500000
2000000
1500000
1000000
500000
0
Number of cells
1 2 3 4 5 6 7 8 9 10
days
mimic Ctrl
mimic miR-378
anti-miR-Ctrl
anti-miR-378

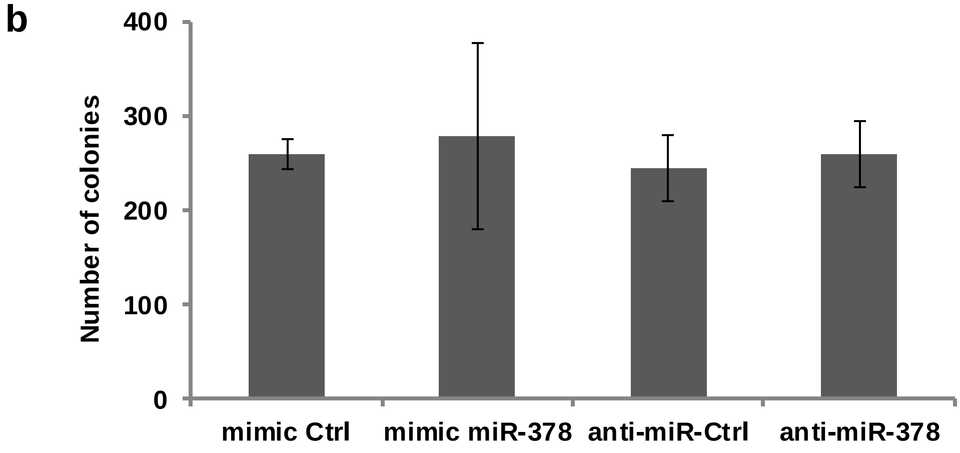
b
400
300
200
100
0
Number of colonies
mimic Ctrl
mimic miR-378
anti-miR-Ctrl
anti-miR-378

Figure S3

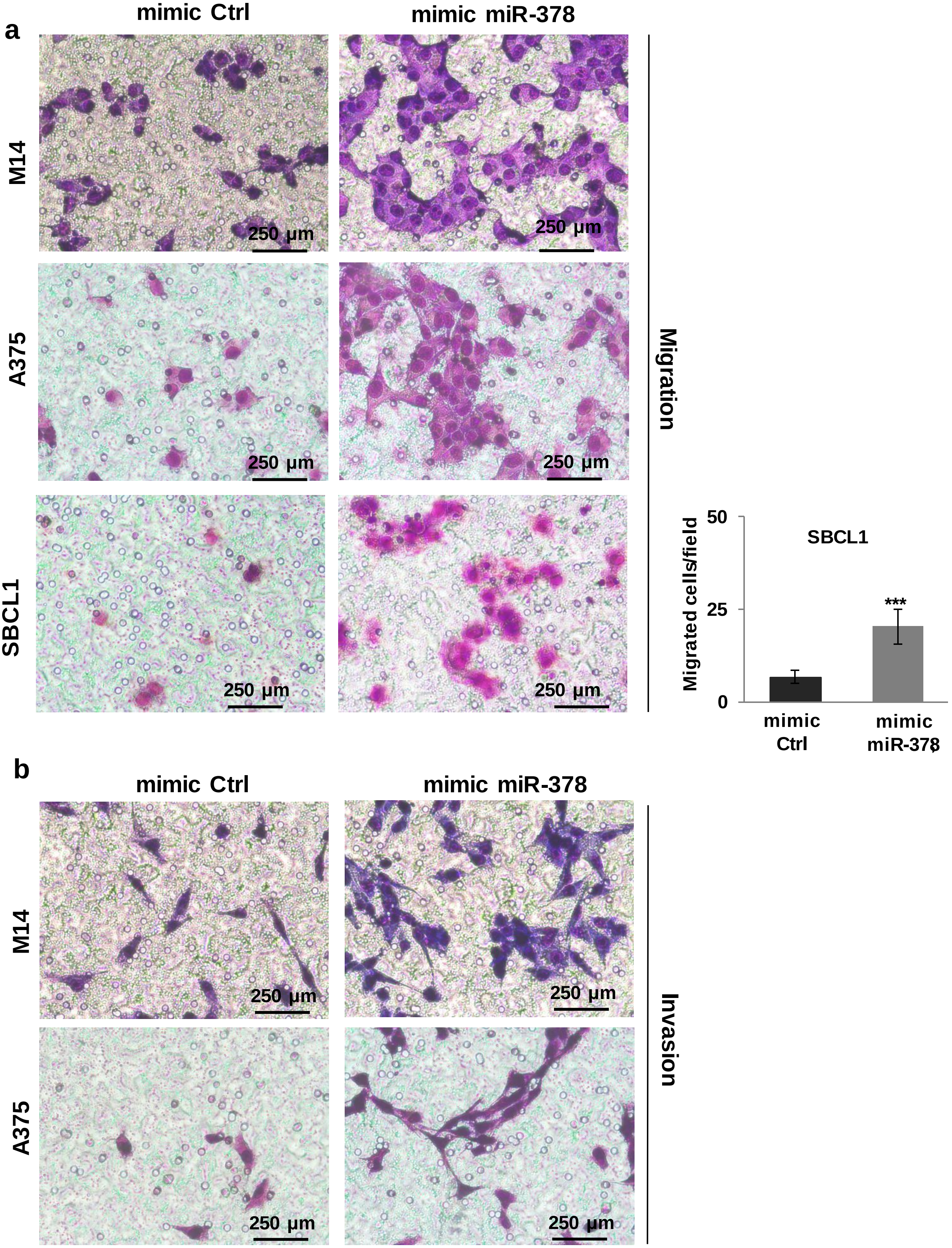
a
mimic Ctrl
mimic miR-378
M14
A375
SBCL1
250 µm
Migration
SBCL1
Migrated cells/field
50
25
0
***
mimic Ctrl
mimic miR-378
b
mimic Ctrl
mimic miR-378
M14
A375
250 µm
Invasion

Figure S4

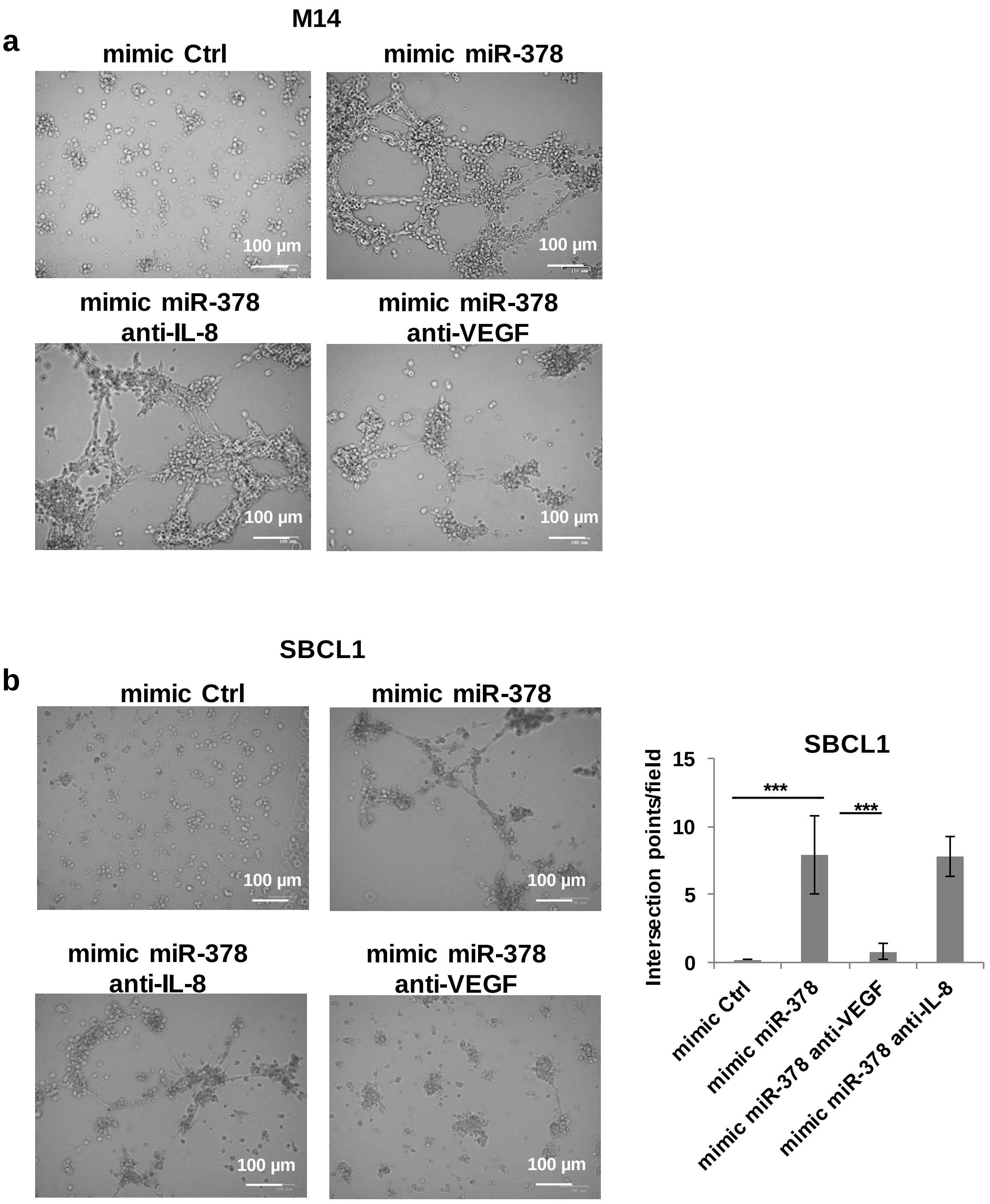
M14
a
mimic Ctrl
mimic miR-378
100 µm
mimic miR-378 anti-IL-8
mimic miR-378 anti-VEGF
SBCL1
b
mimic Ctrl
mimic miR-378
mimic miR-378 anti-IL-8
mimic miR-378 anti-VEGF
SBCL1
Intersection points/field
15
10
5
0
***
***
mimic Ctrl
mimic miR-378
mimic miR-378 anti-VEGF
mimic miR-378 anti-IL-8

Figure S5

a

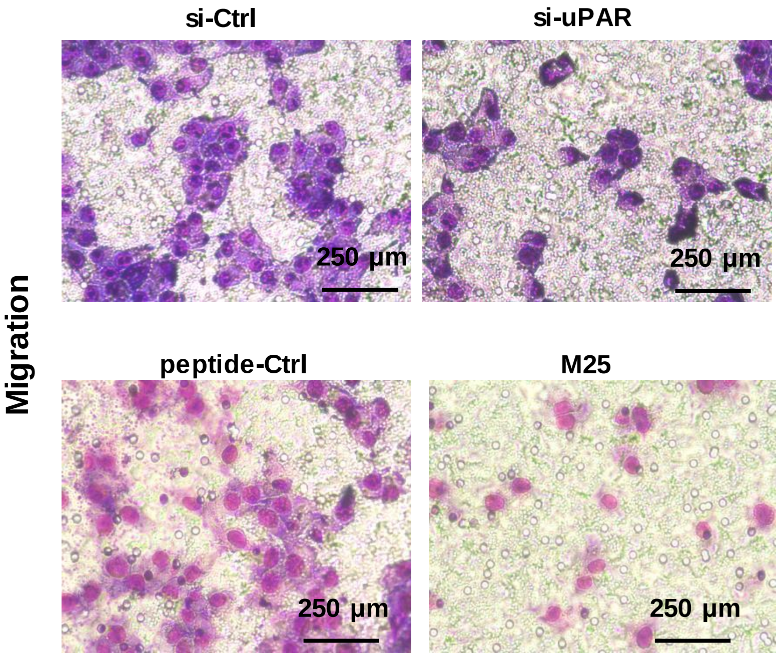
Migration
si-Ctrl
si-uPAR
250 µm
250 µm
peptide-Ctrl
M25
250 µm
250 µm

b

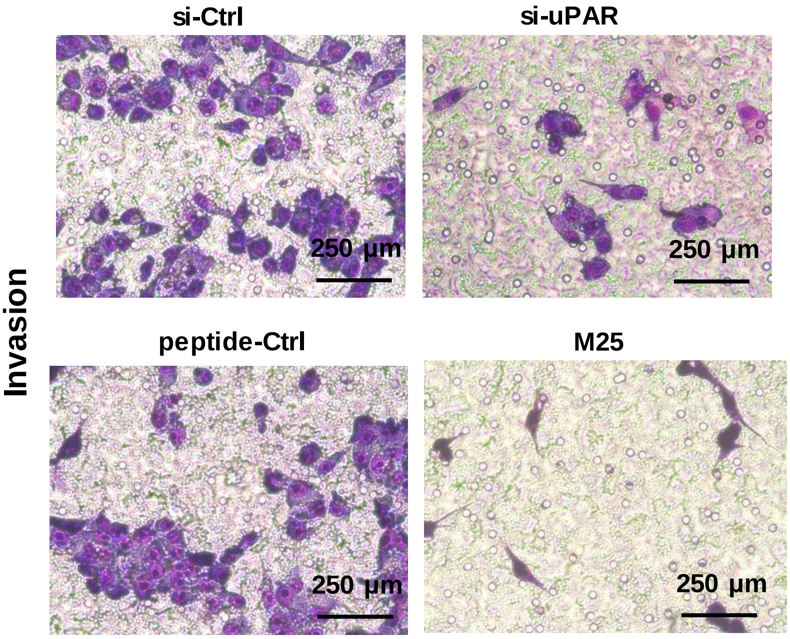
Invasion
si-Ctrl
si-uPAR
250 µm
250 µm
peptide-Ctrl
M25
250 µm
250 µm

Figure S6

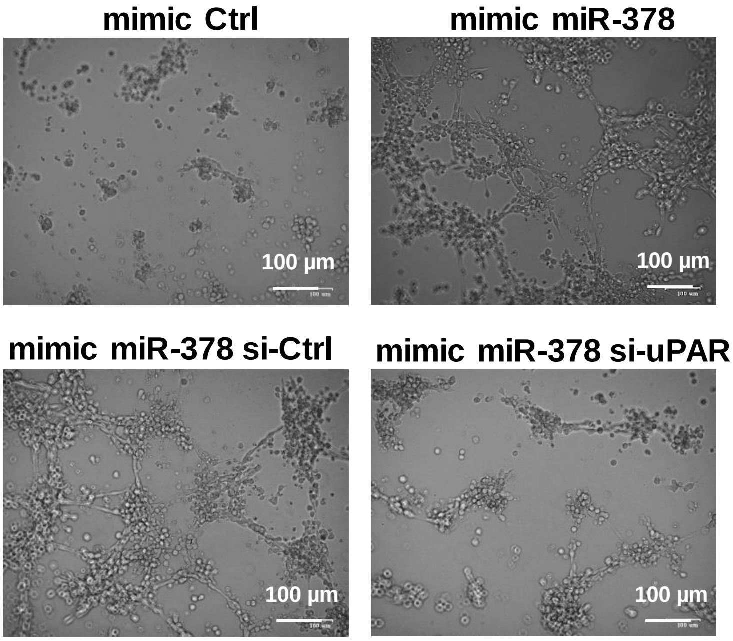
mimic Ctrl
mimic miR-378
100 μm
100 μm
mimic miR-378 si-Ctrl
mimic miR-378 si-uPAR
100 μm
100 μm

Figure S7

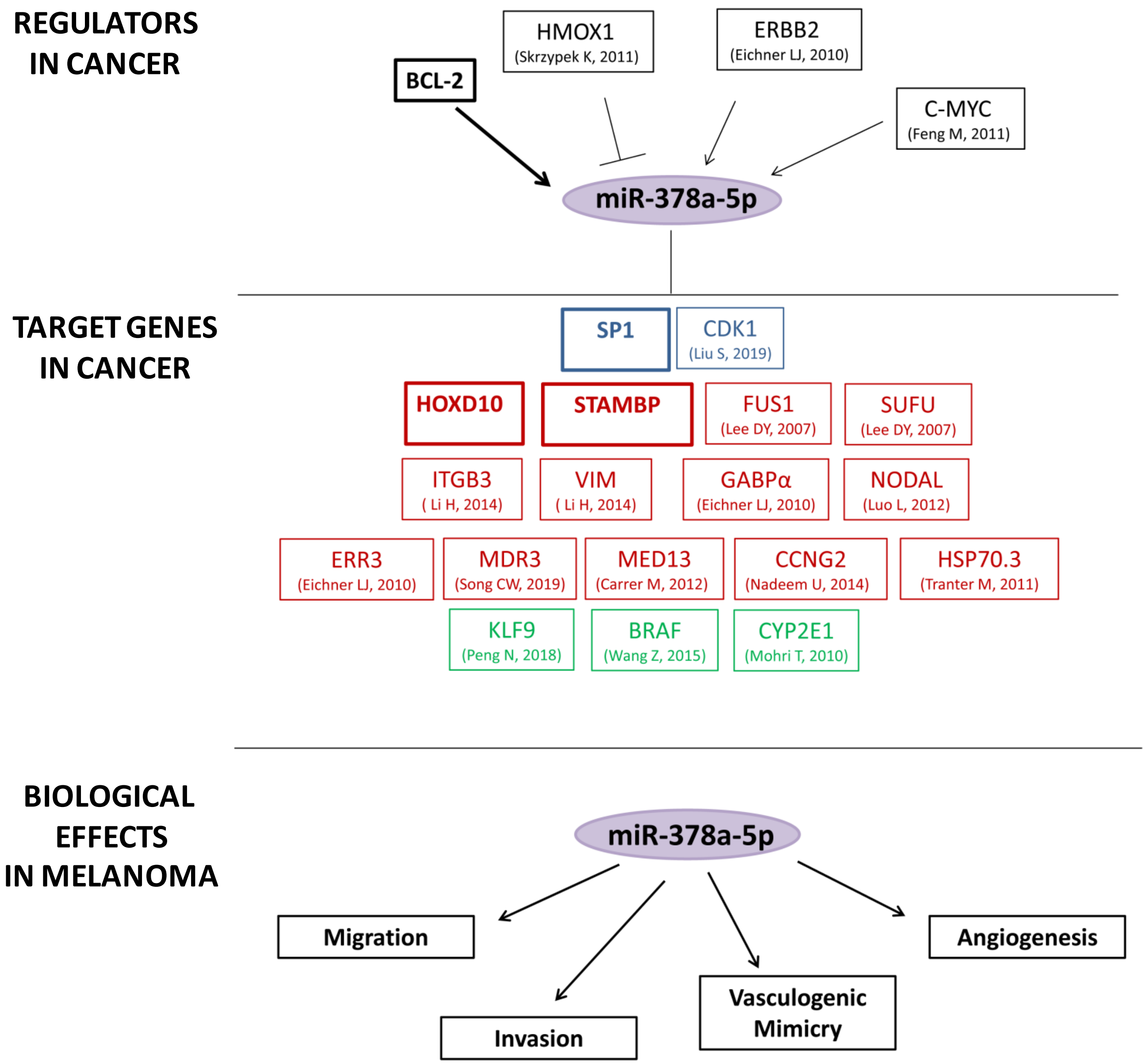
REGULATORS IN CANCER
BCL-2
HMOX1
(Skrzypek K, 2011)
ERBB2
(Eichner LJ, 2010)
C-MYC
(Feng M, 2011)
miR-378a-5p
TARGET GENES IN CANCER
SP1
CDK1
(Liu S, 2019)
HOXD10
STAMBP
FUS1
(Lee DY, 2007)
SUFU
(Lee DY, 2007)
ITGB3
(Li H, 2014)
VIM
(Li H, 2014)
GABPα
(Eichner LJ, 2010)
NODAL
(Luo L, 2012)
ERR3
(Eichner LJ, 2010)
MDR3
(Song CW, 2019)
MED13
(Carrer M, 2012)
CCNG2
(Nadeem U, 2014)
HSP70.3
(Tranter M, 2011)
KLF9
(Peng N, 2018)
BRAF
(Wang Z, 2015)
CYP2E1
(Mohri T, 2010)
BIOLOGICAL EFFECTS IN MELANOMA
miR-378a-5p
Migration
Invasion
Vasculogenic Mimicry
Angiogenesis
